# Supplementary material for: Biomarkers in a socially exchanged fluid reflect colony maturity, behavior, and distributed metabolism
Source: eLife. 2021 Nov 2;10:e74005. doi: 10.7554/eLife.74005 (PMC8608388; doi:10.7554/eLife.74005)
Supplement: Figure 2—source data 1. [file elife-74005-fig2-data1.pdf]

Figure 2 - source data 1: Coefficient of variation by sample type

Post-hoc comparisons of gamma GLM on coefficient of variation by sample type.

Comparison	Estimate	Std. Error	z value	Pr(>|z|)	
ReplicaN-ReplicaL	0.195257	0.036215	5.392	<0.001	
Mature-ReplicaL	0.787579	0.035195	22.377	<0.001	
Young-ReplicaL	0.729474	0.035060	20.807	<0.001	
Lab2020-ReplicaL	0.363673	0.036394	9.993	<0.001	
Lab2019-ReplicaL	0.197683	0.038955	5.075	<0.001	
Nurse-ReplicaL	0.499185	0.035416	14.095	<0.001	
Forager-ReplicaL	0.437798	0.037355	11.720	<0.001	
Mature-ReplicaN	0.592322	0.033084	17.904	<0.001	
Young-ReplicaN	0.534217	0.032939	16.218	<0.001	
Lab2020-ReplicaN	0.168416	0.034356	4.902	<0.001	
Lab2019-ReplicaN	0.002426	0.037059	0.065	1.00000	NS
Nurse-ReplicaN	0.303928	0.033319	9.122	<0.001	
Forager-ReplicaN	0.242541	0.035372	6.857	<0.001	
Young-Mature	-0.058105	0.031815	-1.826	0.60026	NS
Lab2020-Mature	-0.423906	0.033279	-12.738	<0.001	
Lab2019-Mature	-0.589896	0.036063	-16.357	<0.001	
Nurse-Mature	-0.288394	0.032208	-8.954	<0.001	
Forager-Mature	-0.349781	0.034328	-10.189	<0.001	
Lab2020-Young	-0.365801	0.033136	-11.039	<0.001	
Lab2019-Young	-0.531791	0.035931	-14.800	<0.001	
Nurse-Young	-0.230289	0.032059	-7.183	<0.001	
Forager-Young	-0.291676	0.034189	-8.531	<0.001	
Lab2019-Lab2020	-0.165989	0.037233	-4.458	<0.001	
Nurse-Lab2020	0.135513	0.033513	4.044	0.00131	
Forager-Lab2020	0.074125	0.035555	2.085	0.42312	NS
Nurse-Lab2019	0.301502	0.036279	8.311	<0.001	
Forager-Lab2019	0.240115	0.038173	6.290	<0.001	
Forager-Nurse	-0.061387	0.034554	-1.777	0.63467	NS
