# Supplementary material for: Biomarkers in a socially exchanged fluid reflect colony maturity, behavior, and distributed metabolism
Source: eLife. 2021 Nov 2;10:e74005. doi: 10.7554/eLife.74005 (PMC8608388; doi:10.7554/eLife.74005)
Supplement: Figure 2—source data 2. [file elife-74005-fig2-data2.pdf]

Figure 2 - source data 2: Protein number by sample type

Post-hoc comparisons of negative binomial GLM on protein number explained by sample type.

Comparison	Estimate	Std. Error	z value	Pr(>|z|)	
Lab18mon-Forager	0.311827	0.143990	2.166	0.3534	NS
Lab6mon-Forager	0.124075	0.088708	1.399	0.8477	NS
MatL-Forager	0.253416	0.122606	2.067	0.4167	NS
MatN-Forager	0.530447	0.121930	4.350	<0.01	
Mature-Forager	0.341734	0.080648	4.237	<0.01	
Nurse-Forager	0.309891	0.083418	3.715	<0.01	
Young-Forager	0.349596	0.083373	4.193	<0.01	
Lab6mon-Lab18mon	-0.187752	0.146878	-1.278	0.8993	NS
MatL-Lab18mon	-0.058411	0.169518	-0.345	1.0000	NS
MatN-Lab18mon	0.218620	0.169030	1.293	0.8935	NS
Mature-Lab18mon	0.029907	0.142156	0.210	1.0000	NS
Nurse-Lab18mon	-0.001936	0.143745	-0.013	1.0000	NS
Young-Lab18mon	0.037769	0.143719	0.263	1.0000	NS
MatL-Lab6mon	0.129341	0.125986	1.027	0.9676	NS
MatN-Lab6mon	0.406372	0.125328	3.242	0.0237	
Mature-Lab6mon	0.217660	0.085698	2.540	0.1660	NS
Nurse-Lab6mon	0.185816	0.088310	2.104	0.3921	NS
Young-Lab6mon	0.225521	0.088267	2.555	0.1603	NS
MatN-MatL	0.277031	0.151229	1.832	0.5791	NS
Mature-MatL	0.088318	0.120447	0.733	0.9956	NS
Nurse-MatL	0.056475	0.122319	0.462	0.9998	NS
Young-MatL	0.096180	0.122288	0.787	0.9932	NS
Mature-MatN	-0.188713	0.119758	-1.576	0.7498	NS
Nurse-MatN	-0.220556	0.121641	-1.813	0.5918	NS
Young-MatN	-0.180851	0.121610	-1.487	0.8017	NS
Nurse-Mature	-0.031843	0.080210	-0.397	0.9999	NS
Young-Mature	0.007862	0.080163	0.098	1.0000	NS
Young-Nurse	0.039705	0.082950	0.479	0.9997	NS
